# Awareness of Cervical Cancer and Attitude Toward Human Papillomavirus and Its Vaccine Among Ghanaians

**DOI:** 10.3389/fonc.2020.01651

**Published:** 2020-09-08

**Authors:** Emmanuel Kwateng Drokow, Liu Zi, Qian Han, Clement Yaw Effah, Clement Agboyibor, Evans Sasu, Gloria Selorm Akpabla, Francis Foli, Kai Sun

**Affiliations:** ^1^Department of Radiation Oncology, Zhengzhou University People's Hospital and Henan Provincial People's Hospital Henan, Zhengzhou, China; ^2^Department of Radiation Oncology, First Affiliated Hospital of Xi'an Jiaotong University, Xi'an, China; ^3^College of Public Health, Zhengzhou University, Zhengzhou, China; ^4^School of Pharmaceutical Sciences, Zhengzhou University, Zhengzhou, China; ^5^Department of Radiotherapy, National Centre for Radiotherapy and Nuclear Medicine, Korle Bu Teaching Hospital, Accra, Ghana; ^6^Department of Internal Medicine, Tianjin Medical University, Tianjin, China; ^7^Department of Internal Medicine, Seventh-Day Adventist Hospital, Takoradi, Ghana; ^8^Department of Haematology, Zhengzhou University People's Hospital and Henan Provincial People's Hospital Henan, Zhengzhou, China

**Keywords:** Ghana, cervical cancer, vaccines, human papillomavirus, awareness

## Abstract

**Background:** Cervical cancer (CC) is the fourth most commonly diagnosed cancer among women. Ghana is a low-middle- income country with annual diagnosed cases of 3,151 and 2,119 deaths. The high prevalence rate of cervical cancer in Ghana is mainly due to ineffective preventive measures and insufficient knowledge about the disease. Therefore, our objective was to evaluate the level of knowledge and awareness of cervical cancer and attitude toward human papillomavirus and its vaccine among Ghanaians.

**Methods:** This descriptive cross-sectional survey on the awareness of cervical cancer and attitude toward human papillomavirus and its vaccine was carried out from March 2019 to February 2020. SPSS v. 23.0 was used in the data analysis. The participants' demographic characteristics, knowledge of cervical carcinoma, human papillomavirus vaccine and HPV, and the likelihood to be vaccinated were represented as percentages and frequencies. The difference between males and females was assessed using the chi-square test. The logistic regression analysis was used to evaluate the relationship of possible related indicators with the willingness to receive the HPV vaccine. A *p* < 0.05 was considered statistically significant.

**Results:** A total of 1,376 participants were involved in the final analysis. Among the 1,376 participants involved in this survey, 1,240 participants (90.1%) representing 456 males (33.1%) and 784 females (57.0%) were aware of the terminology “cervical cancer” with a significant *p* = 0.001. When stratified by gender, women had significantly greater knowledge, compared to men in terms of “cervical cancer being common in middle age (35–50) females” (75.5 vs. 67.5%, respectively, *p* ≤ 0.001). When stratified by gender, women had significantly greater knowledge of human papillomavirus (54.5 vs. 43.6%, respectively, *p* < 0.001) and the human papillomavirus vaccine (39.3 vs. 33.1%, respectively, *p* = 0.019) compared to men.

**Conclusion:** Majority of the respondents had poor knowledge regarding cervical cancer risk factors, symptoms, HPV, and its vaccine. Hence, this indicates a wakeup call for government to increase the awareness and knowledge level via the media and health professionals.

## Introduction

Carcinoma of the cervix (CC) is the fourth most commonly diagnosed cancer among women with an annual new registered case of 569,847 and 311,365 deaths worldwide (1, 2). Human papillomaviruses (HPV) have been shown to be one of the most common pathogens transmitted through sexual contact in the cervix, and chronic infections of the cervix with high-risk human papillomavirus is required before cervical cancer can develop or occur (3). The HPV-18 and HPV-16 genotypes cause about 70% of the worldwide cervical cancer cases (4). When measured/estimated by sites, the cervix accounts for about 90% of human papillomavirus attributable global cancers, with two-thirds of that occurring in low and middle-income nations (5). This is primarily attributed to a lack of health insurance coverage in screening programs and a well-established nationwide screening system. Nevertheless, the WHO guide on the control of cervical cancer stated that the success of cervical cancer prevention and control mainly depends on cervical carcinoma screening programs (CCSP) and human papillomavirus vaccinations (6). The highest morbidity rates of cervical cancer were recorded in South–Eastern and South Central Asia, South-America, and sub-Saharan Africa (7). The age-standardization rates (ASR) per 100,000 women annually in West-Africa vary from 53.6 in Guinea, 39.5 in Ghana, 33.0 in Nigeria, 30 in Togo, and 28.6 in Burkina Faso in comparison to the 15.2 globally (8). The level of awareness and knowledge of cervical cancer etiology and HPV vaccination in women, to a great extent, influences their participation in screening and vaccination programs. Ghana is a low-middle-income country with annual diagnosed cases of 3,151 and 2,119 deaths, according to the 2018 ICO/IARC summary reports (9). These statistics, however, are prone to underestimate the actual nation's disease burden, as there exist imparity in the event of cervical cancer screening for females with different geographical and demographical indicators across the nation (10). In addition, most women in rural areas may not avail themselves during cervical cancer screening and HPV vaccination due to lack of knowledge, insufficient funding for health service, and high poverty rates (11). It has been generally acknowledged that health disparities are largely influenced by sociodemographic factors like welfare, unemployment, education, social and health care services, work environment, housing, and living (12). The high uptake and effective implementations of the HPV vaccines depend on the general public comprehension of HPV infections, and their ability to understand the efficacy of the HPV vaccines in preventing cervical cancer (13–16). Some studies have shown that encouragement from close relatives can influence the participation of women in cervical cancer screening and consenting to the children's vaccination. Ndejjo et al. (17) reported that Ugandan women who knew someone who had previously participated in the screening program would avail themselves to be screened. Furthermore, Anyebe et al. (18) and Cunningham et al. found that the willingness of women participating in cervical cancer screening was influenced by their husbands' or partners' decision in helping or encouraging them (18, 19). White et al. (20) also reported that most women in Zambia discuss their screening decisions with their close relatives or people within their immediate social circle. These pieces of evidence indicate that women who receive encouragement from their family, friends, or partners are more likely to participate in the screening program. Studies by Chao et al. (21) and Spencer et al. (22) also demonstrated the effect of women's attitudes in relation to human papillomavirus vaccination uptake in their children. They observed that daughters whose mothers undertook screening were more prone to get the vaccination than those whose mothers did not test or wanted to avoid screening personally. Hence, it was realistic to conclude that females who undergo screening are more willing to have their children vaccinated. Additionally, variables like cultural and religious values were reported to affect health practices. Modibbo et al. (23) noticed that those religious beliefs were a barrier to cervical screening. Consequently, a study by Masika et al. (24) discovered that certain religious beliefs were against vaccination. Past studies on HPV in Ghana focused on the prevalence rate and genotype. Domfeh et al. (25) reported a prevalence rate of 10.7% in 75 women seen in the outpatient department. Yar et al. (26) also reported a prevalence rate of 76.6% in 107 women who tested HIV negative and 42.0% in 100 women who tested HIV positive. Thus, no study has been conducted on the awareness of HPV and its vaccines. Furthermore, given that Cervarix vaccine has been introduced in Ghana, research on the perception of women in relation to human papillomavirus vaccinations are extremely important to assess the effects of past educational programs and further aid the decision-making process to promote these HPV vaccines. We, therefore, conducted this study to assess cervical cancer and HPV awareness, HPV vaccine, and the readiness of both men and women to receive these vaccinations since HPV can cause throat cancer, anal cancer, and genital warts in men. We hypothesized that females who are aware of cervical cancer are most likely to participate in vaccination and screening programs. We also hypothesized that religious beliefs have negative influence on the willingness to receive HPV vaccination.

## Materials and Methods

### Study Population

This descriptive cross-sectional survey on the awareness of cervical cancer and attitude toward human papillomavirus and its vaccine was carried out from March 2019 to February 2020. The study population included (i) a Ghanaian resident, either male or female, (ii) must be 18 years and above, (iii) not deaf and dumb, and (iv) women with no history of HPV vaccination. The target population of men and women mostly resided in either Accra, Kumasi, or Takoradi. These three cities were chosen due to their population density and the availability of cervical cancer screening programs. The questionnaires were designed after a comprehensive review of literature from past studies and then approved by experts (27–29). The soundness and legitimacy of the questionnaire were further verified by a review panel of two experts each in oncology, gynecology and obstetrics, and research methodology prior to the pilot survey. Three questions associated with symptoms and signs were modified, and two questions not related to the topic were deleted according to the comments from the expert. Afterwards, a pilot study was conducted with 30 respondents on the pre-final template to determine the questionnaire's clarity. Findings from the current and pilot study demonstrated that Cronbach's alpha was >0.70. Cronbach's alpha evaluates the internal reliability or consistency of a given dataset. The questionnaire-based survey was undertaken after all participants had given written consent, with their anonymity, and confidentiality maintained. The exclusion criteria included women diagnosed with cervical cancer, women with some gynecological condition, and participants who did not provide their consent. The sample size was determined using the minimum sample size formula; thus, “*n* = *Z*^2^ *P*(1 – *P*)/*d*^2^; where, *n* = sample size; *Z* = *z* statistic for a level of confidence. For the level of confidence of 95%, which is conventional, the *Z*-value is 1.96. *P* = expected prevalence or proportion (in proportion of one; if 46%, *P* = 0.46), and *d* = precision (in proportion of one, if 5%, *d* = 0.05)” (16). The calculated sample size was 382 using an expected proportion or prevalence (p) of 46%; *P* = 0.46 (13), considering a 95% confidence interval (CI) and a 5% marginal error. To cover for heterogeneity in the targeted population and further ensure that maximum responses were received, we increased the sampling size and targeted about 1,500 participants. Simple random sampling was used to attain the targeted sample size.

### Data Collection

The selection of group and the designing of our questionnaire was based on (30) Theory of Triadic Influence (TTI) and McLeroy et al. (31) Social Ecological Model (SEM). The Theory of Triadic Influence considers a “3 × 3 frameworks with environmental streams of influence, interpersonal, and intrapersonal crossed by proximal, distal, and ultimate levels of influence.” The Social-Ecological Model (SEM) considers public policy, community, institutional, interpersonal, and intrapersonal as levels of influence for health-related attitudes. Many theoretical concepts are shared by these frameworks, even though these frameworks differ in variable and structure interaction thus, integrating them in this study. Each question in this survey was adapted and modified from previously published articles, and experts' opinions and was written in English in clear and straightforward language.

To aid the respondents answer the questions easily and quickly, the questions covered in the questionnaires, was categorized into sociodemographic, knowledge on cervical cancer, knowledge of HPV vaccine and HPV, the willingness to receive the HPV vaccination themselves and also having their children vaccinated, the rationale for not being willing to be vaccinated and the acceptability to pay for the human papillomavirus vaccination by themselves and interview quality evaluation. The cervical cancer section was subcategorized into (a) knowledge about cervical cancer, (b) knowledge about cervical cancer symptoms, and (c) knowledge about risk factors of cervical cancer. Knowledge of cervical cancer was evaluated if a participant responded that they were aware of cervical carcinoma by stating that one has heard or knows about cervical cancer. The participant's knowledge regarding the risk factors (“Can HPV infection cause cervical cancer,” “long term use of oral contraceptives pills,” “smoking,” “unprotected sexual practices,” “multiparity,” “Immunocompromised/HIV-AIDS,” “early age at marriage”) and symptoms (“lower abdominal pain,” “bleeding after sexual intercourse,” “bleeding in between periods,” “vaginal discharge with foul smell,” “weight loss,” “post-menopausal bleeding,” and “asymptomatic”) of cervical carcinoma was evaluated. A 30-point score was used to evaluate cervical cancer knowledge. Five points were allocated to every sub-section of knowledge; hence participants were required to range between 0 and 30 scores. One point was allocated to each true response, and zero points to the incorrect response. Participants who responded only “Yes” to the questionnaire's first statement, “Do you know about cervical cancer?” were assigned a knowledge score. Participants' level of knowledge while calculating the knowledge score was categorized using Bloom's cut off point (32). Participants who had from 24 to 30 point were regarded as having excellent knowledge with right answers of 80–100%, participants who scored from 18 to 23 point were regarded as having moderate knowledge with right answers of 60–79%, and participants who scored <18 points were lastly regarded as having poor knowledge with right answers of less than 60%. Information on cervical carcinoma was given to all participants to bridge the knowledge disparity after the end of the cervical cancer sub-section.

HPV awareness was evaluated with the phrase, “Have you heard of HPV?” Participants who responded “yes” to this statement were regarded to have knowledge about HPV. The knowledge on HPV vaccine was evaluated in the same manner. Some previous studies have reported these questions (29). Other relevant questions such as, “Is HPV infection a sexually transmitted infection?,” “Is a persistent infection of high-risk HPV the leading cause of cervical cancer and other HPV cancer types?,” “Can the HPV vaccine prevent cervical cancer and other HPV cancer types?,” and “Must the HPV vaccination be received before the first sexual intercourse?” were preliminary used in evaluating participants' knowledge concerning human papillomavirus and its vaccine. Similar questions used by past studies (28) in evaluating the HPV vaccination acceptability by asking, “Are you willing to vaccinate your current or future children both male and female?,” “Are you willing to vaccinate yourself?,” and “Will you accept that you pay for the HPV vaccination by yourself?” were also used in this study. Specific questions contained three possible outcomes (don't know, no, yes); however, the “don't know” option was regarded as an incorrect response.

### Data Analysis

SPSS v. 23.0 was used in the data analysis. The participants' demographic characteristics, knowledge of cervical carcinoma, human papillomavirus vaccine and HPV, and likelihood to be vaccinated were represented by percentages and frequencies. The difference between males and females was assessed using the chi-square test. The logistic regression analysis was used to evaluate the relationship of possible related indicators with the willingness to receive the HPV vaccine. Indicators in the univariate model were integrated into a multivariate logistic regression, in which confidence intervals of 95% and the adjusted odds ratio were estimated. A stratified assessment was conducted to determine whether gender affected the factors correlated with the willingness to be vaccinated. A *p* < 0.05 was considered statistically significant.

## Results

### Sociodemographic Characteristics

Of the total 1,500 survey respondents, 124 answered the questionnaires with inconsistent and incomplete responses. After eliminating the inconsistent and incomplete questionnaires, the remaining questionnaires were analyzed and the total response rate was 91.73%. A total of 1,376 participants were involved in the final analysis. Table 1 represents the sociodemographic characteristic of the participants. The participants' mean age was 35.5 [Standard Deviation (SD) ±6.4] years. A total of 532 (38.7%) were males, and the remaining 844 (61.3%) were females. Among them, 1,316 (95.6%; males = 496, females = 820) were Christians. The proportions of ethnicity based on Akan, Ewe, Ga, and others were 71.8, 12.8, 7.3, and 8.1%, respectively. Five-point eight percent of the participants had been educated at the senior high school level and below. Sixteen-point, six percent of the respondents, were not on any insurance policy, and 46.8% had a monthly income of <2,000 Ghana cedis equivalent to $350. Fifty-one-point, 1% of the respondents, were working, and 39.5% were students. Majority of the respondents were single (86.9%) and 61.0% (males = 292, females = 548) had their first sexual intercourse at age 18 years old and above with 47.4% (males = 220, females = 432) having only “one sexual partner in the past 6 months.” Statistical significance was noticed in most of the sociodemographic variables except medical insurance, marital status, and age.

**Table 1:** Sociodemographic characteristics of participants.

**Sociodemographic**	**Gender**		**Chi-square**	***p*-value**
**characteristics**				
	**Male**	**Female**		
	**(*N* = 532)**	**(*N* = 844)**		
**Age**				
<40	508 (95.5)	804 (95.3)		
40–60	20 (3.8)	32 (3.8)	0.142^a^	0.974^a^
Above 60	4 (0.8)	8 (0.9)		
**Tribe**				
Akan	396 (74.4)	592 (70.1)		
Ewe	64 (12.0)	112 (13.3)	11.127^b^	0.011^b^
Ga	24 (4.5)	76 (9.0)		
Others	48 (3.5)	64 (4.7)		
**Religion**				
Christian	496 (93.2)	820 (97.2)		
Muslim	28 (5.3)	20 (2.4)	14.472^a^	0.001^a^
Traditionalist	4 (0.8)	4 (0.5)		
Others	4 (0.8)	0 (0)		
**Education**				
Junior high school or below	4 (0.8)	4 (0.5)		
Senior high school	20 (3.8)	48 (5.7)	5.246^a^	0.133^a^
College/graduate and above	508 (95.5)	788 (93.4)		
Not applicable	0	4 (0.5)		
**Occupation**				
Student	232 (43.6)	312 (37.0)		
Working	252 (47.4)	452 (53.6)	11.215^b^	0.011^b^
Retired	12 (2.3)	8 (0.9)		
Unemployed	36 (6.8)	72 (8.5)		
**Marital status**				
Single/divorced/widow	468 (88.0)	728 (86.3)	0.843^b^	0.359^b^
Married	64 (12.0)	116 (13.7)		
**Medical Insurance**				
No insurance	104 (19.5)	124 (14.7)		
NHIS	364 (68.4)	612 (72.5)	5.716^b^	0.126^b^
Commercial Insurance	28 (5.3)	44 (5.2)		
Company Insurance	36 (6.8)	64 (7.6)		
**Age at sex debut (year)**				
<18	76 (14.3)	72 (8.5)		
>18	292 (54.9)	548 (64.9)	27.567^b^	<0.001^b^
Don't know	48 (9.0)	36 (4.3)		
None	116 (21.8)	188 (22.3)		
**Age at menarche (year)**				
<12	0	144 (17.1)		
>12	0	660 (78.2)	1,813.155^a^	<0.001^a^
Unknown	0	40 (4.7)		
Not applicable for male	532	0		

a *Fisher's exact analysis was performed for tables which had at least one expected value <5 in the cells*.

b *Pearson Chi-square test was performed for tables with 0 expectant cell count. The color values means Fisher's exact analysis was used*.

### Knowledge About Cervical Cancer

Among the 1,376 participants involved in this survey, 1,240 participants (90.1%) representing 456 males (33.1%) and 784 females (57.0%) were aware of the terminology “cervical cancer.” When stratified by gender, women had significantly greater knowledge, compared to men in terms of “cervical cancer being common in middle age (35–50) females” (75.5 vs. 67.5%, respectively, *p* ≤ 0.001). These participants were examined further to test their knowledge on some risk factors and symptoms of cervical cancer, as presented in Tables 2–4. Majority of the respondents were aware of “bleeding after sexual intercourse (correctly identified by 51.8% of men and 70.4% of women, *p* ≤ 0.001),” “lower abdominal pain (correctly identified by 59.6% of men and 71.9% of women, *p* ≤ 0.001)” and “vaginal discharge with foul smell (correctly identified by 61.4% of men and 68.4% of women, *p* ≤ 0.001)” as being the dominant cervical cancer symptoms. Likewise, a high proportion among the responses regarding the risk factors “Human papillomavirus infection (correctly identified by 47.4% of men and 55.6% of women, *p* = 0.010)” and “unprotected sexual practices (correctly identified by 50.9% of men and 63.8% of women, *p* ≤ 0.001)” was noticed. Our survey respondents were ranked in each sub-category according to their level of knowledge in cervical cancer epidemiology, symptoms, and risk factors. In general, 75.3% of the respondents had good knowledge of cervical carcinoma epidemiology; however, it was accompanied by moderate knowledge in terms of cervical cancer risk factors (63.8%) and symptoms (61.6%) per the Bloom's cut-off point for accessing knowledge level. Respondents were asked regarding sources of information on cervical carcinoma and the main sources were social media/radio/television (*N* = 851, 68.8%), nurses/doctors (*N* = 507, 40.9%), newspapers/magazines (*N* = 255, 20.7%), and relatives/family (*N* = 221, 17.8%).

**Table 2:** Knowledge on cervical cancer epidemiology.

**Variable**	**Gender**		**Chi-square**	***p*-value**
	**Male**	**Female**		
	**(*N* = 532)**	**(*N* = 844)**		
**Do you know about cervical cancer?**				
Yes	456 (85.7)	784 (92.9)	18.87	<0.001
No	76 (14.3)	60 (7.1)		
**Is cervical cancer a communicable disease (transmitted by skin contact**,				
**sneezing, and coughing)^a^**				
Yes	16 (3.5)	16 (2.0)	39.221	<0.001
No	364 (79.8)	720 (91.8)		
Don't know	76 (16.7)	48 (6.1)		
**Is cervical cancer more common in middle age females?^a^**				
Yes	324 (71.1)	668 (85.2)	36.086	<0.001
No	125 (27.4)	106 (13.5)		
Don't know	7 (1.5)	10 (1.3)		
**Are all women at risk of developing cervical cancer?^a^**				
Yes	308 (67.5)	592 (75.5)	18.989	<0.001
No	68 (14.9)	120 (15.3)		
Don't know	80 (17.5)	72 (9.2)		
**Is cervical cancer more common in middle age females?^a^**				
Yes	264 (57.9)	584 (74.5)	39.221	<0.001
No	28 (6.1)	48 (6.1)		
Don't know	164 (36.0)	152 (19.4)		

*Values are presented as number (%). The chi-square test was used and p < 0.05 was considered as statistically significant*.

a *Only participants who have heard of cervical cancer answered these questions*.

**Table 3:** Knowledge on cervical cancer symptoms answered by only participants who have heard of cervical cancer.

**Symptoms**	**Gender**		**Chi-square**	***p*-value**
	**Male**	**Female**		
	**(*N* = 456)**	**(*N* = 784)**		
**Asymptomatic (no symptoms)**				
Yes	96 (21.1)	252 (32.1)	18.75	<0.001
No	136 (29.8)	220 (28.1)		
Don't know	224 (49.1)	312 (39.8)		
**Post-menopausal bleeding**				
Yes	200 (43.9)	424 (54.1)	15.315	<0.001
No	20 (4.4)	44 (5.6)		
Don't know	236 (51.8)	316 (40.3)		
**Weight loss**				
Yes	180 (39.5)	420 (53.6)	23.226	<0.001
No	56 (12.3)	80 (10.2)		
Don't know	220 (48.2)	284 (36.2)		
**Vaginal discharge with foul smell**				
Yes	280 (61.4)	536 (68.4)	6.739	<0.001
No	32 (7.0)	52 (6.6)		
Don't know	144 (31.6)	196 (25.0)		
**Bleeding in between periods**				
Yes	212 (46.5%)	484 (61.7)	29.448	<0.001
No	20 (4.4)	36 (4.6)		
Don't know	224 (49.1)	264 (33.7)		
**Bleeding after sexual intercourse**				
Yes	236 (51.8)	552 (70.4)	62.377	<0.001
No	20 (4.4)	52 (6.6)		
Don't know	200 (43.9)	180 (23.0)		
**Lower abdominal pain**				
Yes	272 (59.6)	564 (71.9)	19.905	<0.001
No	32 (7.0)	36 (4.6)		
Don't know	152 (33.3)	184 (23.5)		

**Table 4:** Knowledge on cervical cancer risk factors answered by only participants who have heard of cervical cancer.

**Risk factors**	**Gender**		**Chi-square**	***p*-value**
	**Male**	**Female**		
	**(*N* = 456)**	**(*N* = 784)**		
**Early age at marriage**				
Yes	88 (19.3)	168 (21.4)	3.544	0.170
No	204 (44.7)	308 (39.3)		
Don't know	164 (36.0)	308 (39.3)		
**Immunocompromised/Human immunodeficiency virus/AIDS**				
Yes	160 (35.1)	276 (35.2)	7.048	0.029
No	128 (28.1)	172 (21.9)		
Don't know	168 (36.8)	336 (42.9)		
**Multiparity (giving birth to more than 3 children)**				
Yes	48 (10.5)	112 (14.3)	8.511	0.015
No	220 (48.2)	316 (40.3)		
Don't know	188 (41.2)	356 (45.4)		
**Unprotected sexual practices**				
Yes	232 (50.9)	500 (63.8)	25.342	<0.001
No	52 (11.4)	92 (11.7)		
Don't know	172 (37.7)	192 (24.5)		
**Smoking**				
Yes	192 (42.1)	388 (49.5)	14.575	0.001
No	40 (8.8)	96 (12.2)		
Don't know	224 (49.1)	300 (38.3)		
**Long term use of oral contraceptives pills**				
Yes	208 (45.6)	396 (50.5)	14.216	0.001
No	28 (6.1)	84 (10.7)		
Don't know	220 (48.2)	304 (38.8)		
**Human papillomavirus (HPV) infection**				
Yes	216 (47.4)	436 (55.6)	9.117	0.010
No	16 (3.5)	32 (4.1)		
Don't know	224 (49.1)	316 (40.3)		

### Knowledge About HPV and Its Vaccine

As presented in Table 5 of the participant who answered the questions, 50.3% (*N* = 692) have “heard of HPV,” and only 36.9% have “heard of the HPV vaccine.” When stratified by gender, women had significantly greater knowledge of human papillomavirus (54.5 vs. 43.6%, respectively, *p* < 0.001) and the human papillomavirus vaccine (39.3 vs. 33.1%, respectively, *p* = 0.019) compared to men.

**Table 5:** Attitude and awareness of HPV and the HPV vaccine among participants.

**Items**	**Total (*N* = 1,376)**	**Male (*N* = 532)**	**Female (*N* = 844)**	***p*-value**
**Have you heard of HPV?**				
Yes	692 (50.3)	232 (43.6)	460 (54.5)	<0.001
No	684 (49.7)	300 (56.4)	384 (45.4)	
**Is HPV infection a sexually transmitted infection?^a^**				
Yes	414 (59.8)	113 (48.7)	301 (65.4)	<0.001
No	278 (40.2)	119 (51.3)	159 (34.6)	
**Is persistent infection of high-risk HPV the leading cause of cervical cancer and other HPV cancer types?^a^**				
Yes	520 (75.1)	155 (66.8)	365 (79.3)	<0.001
No	172 (24.9)	77 (33.2)	95 (20.7)	
**Have you heard of the HPV vaccine?**				
Yes	508 (36.9)	176 (33.1)	332 (39.3)	<0.001
No	868 (63.1)	356 (66.9)	512 (60.7)	
**Can the HPV vaccine prevent cervical cancer and other HPV cancer types?^b^**				
Yes	284 (55.9)	80 (45.5)	204 (61.4)	<0.001
No	224 (44.1)	96 (54.5)	128 (38.6)	
**Must the HPV vaccination be received before the first sexual intercourse?^b^**				
Yes	110 (21.7)	52 (29.5)	58 (17.5)	0.001
No	398 (78.3)	124 (70.5)	274 (82.5)	
**Are you willing to vaccinate yourself?**				
Yes	1,108 (80.5)	352 (66.2)	756 (89.6)	<0.001
No	268 (19.5)	180 (33.8)	88 (10.4)	
**Are you willing to vaccinate your current or future children both male and female?^c^**				
Yes	900 (81.2)	266 (75.6)	634 (83.9)	<0.001
No	208 (18.8)	86 (24.4)	122 (16.1)	
**What are your reasons for unwillingness to take the HPV vaccine**				
Worry about the safety	81 (30.2)	38 (21.1)	43 (48.9)	0.001
The HPV vaccine has not been widely accepted	35 (13.1)	23 (12.8)	12 (13.6)	
Worry about the price	35 (13.1)	15 (8.3)	20 (22.7)	
Worry about the effectiveness	34 (12.7)	28 (15.6)	6 (6.8)	
Not considering themselves at risk of cervical cancer	30 (11.2)	27 (15.0)	3 (3.4)	
The vaccine is not protective	28 (10.4)	26 (14.4)	2 (2.3)	
Other reasons	25 (8.6)	23 (12.8)	2 (2.3)	
**Will you accept that you pay for the HPV vaccination by yourself?^d^**				
Yes	331 (29.9)	145 (41.2)	186 (24.6)	<0.001
No	777 (70.1)	207 (58.8)	570 (75.4)	

*Values are presented as number (%). The chi-square test was used and p <0.05 was considered as statistically significant*.

a *Participants who have heard of human papillomavirus (HPV) answered these questions*,

b *Participants who have heard of the HPV vaccine answered these questions*,

c *Participants who are not willing to take the HPV vaccine answered the question*,

d *Participants who are willing to take the HPV vaccine answered the question*.

Among the respondents who have heard of HPV, 59.8% (*N* = 414) of the respondents were aware that HPV infection is transmitted through sexual contact (correctly identified by 48.7% of men and 65.4% of women, *p* < 0.001) and 75.1% of the respondents were aware that “the persistent infection of high-risk HPV is the leading cause of cervical cancer and other HPV cancer types” with a significant *p* < 0.001. Furthermore, among respondents with knowledge of the HPV vaccine, only 55.9% (*N* = 284, *p* < 0.001) knew that cervical cancer could be prevented with the HPV vaccine. Additionally, only 21.7% (*N* = 110, *p* = 0.001) knew that “HPV vaccination is needed before first sexual intercourse.”

In general, 80.5% (*N* = 1,108) respondents were willing to have the HPV vaccination. Likewise, women had significantly greater willingness, compared to men (89.6 vs. 66.2%, respectively, *p* < 0.001). A total of 83.9% of women were willing to vaccinate their current and future children. Furthermore, the major reasons for respondents refusing to undertake the HPV vaccinations were “worrying about the safety of vaccine (30.2%),” “the HPV vaccine has not been widely accepted (13.1%),” “worrying about the price (13.1%),” and “worry about the effectiveness (12.7%).” Participants who said no to the payment for the HPV vaccine suggested that, WHO (70.4%) and the government (60.5%) should ensure the free supply of the HPV vaccine.

### Willingness to Receive HPV Vaccine and Its Associated Factors

The bivariate regression analysis demonstrated a significant relationship exists between the willingness to be vaccinated and age, religion, economic status, education, age at first sex, participants with knowledge of cervical, HPV and its vaccine. This finding showed age 18–35 years (OR = 1.475; 95%CI = 1.142–1.591), respondents who are Christians (OR = 1.275; 95%CI = 0.729–1.459), college/graduate students (OR = 1.218; 95%CI = 1.054–1.878), respondents who had their first sex at age above18 years (OR = 1.670; 95%CI = 1.484–1.929), respondents with monthly income 2,000–3,999 Ghana cedis (OR = 1.686; 95%CI = 1.136–2.501), respondent with knowledge about CC (OR = 0.541; 95%CI = 0.364–0.803), respondent who have heard of HPV (OR = 0.760; 95%CI = 0.581–0.993) and heard of HPV vaccine (OR = 0.870; 95%CI = 0.657–1.150) were more willing to receive the HPV vaccinations. Hence, a strong association between these variables and a respondent willingness to be vaccinated. Table 6 shows the outcome of the univariate and multivariate logistics analysis.

**Table 6:** Variables associated with the willingness to receive HPV.

**Variables**	**Willingness to receive HPV vaccination**	**Willingness to receive HPV vaccination**
	**Odd Ratio 95% Confidence Interval**	**Adjusted odd ratio 95%Confidence Interval**
**Gender**		
Male	1	**1**
Female	0.228 (0.171–0.303)	0.423 (0.272–0.504)
**Age**		
18–35	1.475 (1.142–1.591)	1.527 (1.177–1.684)
36–60	0.600 (0.154–2.344)	0.547 (0.121–2.412)
Above 60	1	1
**Tribe**		
Akan	1.639 (0.945–2.843)	1.862 (1.218–2.945)
Ewe	0.769 (0.380–1.557)	0.804 (0.430–1.752)
Ga	1.500 (0.729–3.085)	1.532 (0.932–3.148)
Others	1	1
**Religion**		
Christian	1.275 (0.729–1.459)	1.672 (1.129–1.814)
Muslim	1.151 (0.523–2.233)	1.407 (0.779–2.476)
Traditionalist	1	1
**Education**		
Junior high school or below	1	1
Senior high school	0.700 (0.700–3.037)	1.056 (1.032–3.193)
College/graduate and above	1.218 (1.054–1.878)	1.431 (1.101–1.922)
**Occupation**		
Student	1.354 (0.801–2.287)	1.453 (0.843–2.356)
Working	0.832 (0.492–1.408)	0.946 (0.563–1.503)
Retired	2.933 (2.933–8.117)	2.965 (2.945–8.271)
Unemployed	1	1
**Marital status**		
Single/divorced/widow	1	1
Married	1.213 (0.830–1.774)	1.275 (0.992–1.806)
**Medical Insurance**		
No insurance	1	1
NHIS	0.676 (0.480–0.952)	0.772 (0.566–1.028)
Commercial Insurance	1.536 (0.864–2.730)	1.653 (0.941–2.804)
Company Insurance	0.419 (0.213–0.822)	0.526 (0.391–0.987)
**Monthly Income (GH Cedis)**		
<2,000	1	1
2,000–3,999	1.686 (1.136–2.501)	1.719 (1.145–2.643)
4,000–5,999	2.205 (1.062–4.577)	2.259 (1.167–4.689)
6,000–9,999	1.102 (0.360–3.378)	1.174 (0.463–3.387)
Above 10,000	2.205 (0.649–7.485)	2.249 (0.754–7.584)
**Age at first sex**		
<18	1	1
>18	1.670 (1.484–1.929)	1.708 (1.526–1.981)
**Number of sexual partners in the past 6 months**		
1	1	1
>2	2.369 (1.549–3.625)	2.532 (1.671–3.732)
**Do you know about cervical cancer?**		
Yes	0.541 (0.364–0.803)	0.758 (0.495–1.205)
No	**1**	1
**Have you heard of HPV?**		
Yes	0.760 (0.581–0.993)	0.928 (0.685–1.112)
No	**1**	1
**Have you heard of HPV vaccine?**		
Yes	0.870 (0.657–1.150)	1.147 (0.973–1.342)
No	1	

*The bold values are reference values*.

## Discussion

Advancement in understanding cervical carcinoma has been effective in acknowledging its preventive nature (33). It is firmly known that effective screening and HPV vaccination, to a large extent, will significantly decrease the prevalence of the disease (33, 34). For effective prophylaxis and screening, it is of paramount significance to understand the beliefs, perceptions, and knowledge of the general public. The assertion was that, females who were aware of cervical carcinoma are most likely to participate in vaccination and screening programs. Our findings confirmed the hypothesis when a participant responded that they know about cervical cancer, HPV, and its vaccine. This was evident in both women and men. Men who know of cervical carcinoma were most likely to offer encouragement to their partners to participate in vaccination and screening programs. The total awareness of study participants was poor, similar to past studies (35). These findings also complement the results of a systematic review, which reported lower knowledge levels of cervical cancer awareness but a higher willingness to receive vaccination in sub-Saharan Africa (36).

Our study findings showed that majority (90.1%) of the participants were knowledgeable of the term cervical cancer which is higher when compared with other similar studies in developing countries such as Pakistan, Ethiopia, and Zambia and where the percentage of participants knowledgeable of the term cervical cancer were 51.3, 76.8, and 36.8%, respectively (29, 37, 38). The variation may be attributable to the dissemination of information through various mass media and the availability of screening programs in Ghana. The knowledge of respondents on cervical cancer showed that 63.8% of participants know of cervical cancer risk factors.

Among these risk factors, “human papillomavirus infection” and “unprotected sexual practices” were correctly and highly identified as cervical cancer risk factors. This finding is lower when compared to past studies conducted in South Africa, Bhutan, Malaysia, and Ukraine (39–42). This lack of awareness normally leads to the higher death rate related with CC because women who are not enlightened about these risk factors will not undertake the appropriate preventive actions.

Regarding awareness of cervical cancer symptoms, respondents were aware of symptoms such as “vaginal discharge with a foul smell,” “bleeding after sexual intercourse,” and “bleeding in-between period” (67.8, 63.5, and 56.1%, respectively). Our results indicate that much effort is needed to educate the general public, especially women of cervical cancer symptoms, since failure to recognize these symptoms or late presentation can results in delaying medical care, resulting in poor prognosis and higher death rates.

There is no doubt that basic knowledge is essential in encouraging women to patronage preventive actions. This is in line with the intrapersonal level regression findings for SEM, where basic knowledge concerning cervical cancer was the key predictor of attitude (38). Communities based educational programs have been shown to be effective in increasing preventive practices, knowledge, and awareness (43). Social media /radio/television, nurses/doctors, and newspapers/magazines were found as reliable information sources and they could offer potential targets for performing interventional studies intended to improve awareness of cervical cancer in Ghana. It was presumed that females who receive backing from close relatives could influence the participation of women in cervical cancer screening. The findings back the assertion about women participating in screening. It was observed that women's recognized that the acceptance of spouses, families, and friends affected their screening practices. This result is consistent with another study conducted in Zambia that assessed that relatives and peers often spurred women's choice to screen (20). Ndejjo et al. (17) reported that Ugandan women who knew someone who had previously participated in the screening program would avail themselves to be screened. Furthermore, Anyebe et al. (18) and Cunningham et al. (19) found that the willingness of women participating in cervical cancer screening was influenced by their husbands' or partners' decisions in helping or encouraging them. In addition, with the exception of men, women would be more prone to have their daughter's vaccinated if they gain approval from their spouses. It can be proposed that there exists an association between women needing support and participating in taking preventive programs. This suggests that the Ghanaian community is a patriarchal community in which men have a significant influence on the households—indicating that men must be included as a target group for the effectiveness of cervical cancer preventive programs.

Religious beliefs were believed to hinder screening and vaccine uptake. The findings contradict this hypothesis in that there was no influence of religion on screening choices, but rather religion had a good impact on the acceptability of vaccination. This is contradictory to other nations where religion was observed to hinder uptake of vaccination (24, 44). About 95.6% of the respondents acknowledged being Christians, and this improved the likelihood of having themselves vaccinated. This indicates that Ghanaian churches can play a part in enhancing vaccinations program in Ghana. The potential reason is that it is known that certain Christian denominations consciously enlighten their members on medical problems such as cervical cancer. These explanations are insufficient since the participant's Christian denominations were not evaluated. Again, beliefs vary from one Church to another. Likewise, the truthfulness of the information given by the churches requires further investigation.

Previous studies on HPV awareness with a large sample size reported that the percentage of women knowledgeable of HPV ranged from 15.0 to 44.9% (45–48). We noticed a greater percentage of 54.5% in only women, while the total percentage among all the respondents was 50.3%. This finding is lower when compared with results from developed nations since the percentage of women knowledgeable of the human papillomavirus was 71.8, 61.6, and 87.7% in Australia, United Kingdom, and the United States, which indicates that women in developed countries might be more knowledgeable of HPV (49). Furthermore, 36.9% of women were aware of the HPV vaccine. This outcome is slightly higher than what was reported in Chinese women by Lin et al. (28) (21.0%). Nevertheless, women's knowledge of the human papillomavirus vaccine is still far from women in economically developed nations where governmentally funded HPV vaccination program has been implemented per WHO recommendation (49). The possible relationship between HPV awareness and its vaccine and socioeconomic characteristics requires further investigation because populations showed a variety of socioeconomic, ethnic, cultural, and other inequalities in published surveys.

Even though there was a higher willingness of participants to accept the vaccination, the primary complaints among participants not willing to accept the vaccination were how safe the vaccine is, in addition to the acceptability of it worldwide and the price of the vaccine. This finding is similar to a study by (50), where participants were willing to undertake the vaccination at a free cost or receives a subsidiary from the government (51). Endarti et al. further showed that the knowledge of vaccine effectiveness increased the willingness of people to undertake vaccination (51). Hence, the government may use the media houses to educate people on the efficacy of the vaccine. In addition, the economic status may play an important part in the acceptance of the vaccine because our study showed that the likelihood of respondents paying for the vaccination was relatively low in the general population sample, which corresponds with the assumption that the majority of the respondents had lower-income rates. However, it was also striking that higher income earners and participant with company insurance were less willing to pay for vaccination and this can be attributable to the fact that majority of the higher income earners have company or private insurance hence their unwillingness to pay for the vaccination but instead, wants the insurance companies to cover the cost for the vaccination. Again, a greater comprehension of how insurance coverage and certain variables influence HPV vaccination uptake is required to allow potential interventions to be planned, therefore our next research will concentrate on this.

In our survey, most Ghanaians claim to be affiliate with the Christian religion. Hence improvement in the vaccine coverage can be increased with clergy's the support since the majority of these churches are mostly arraigned of refusing western medication due to biblical and moral values. Again, regular visitation to Church must be taken to motivate church members to participate in vaccination programs and further ensure that accurate information on cervical cancer is disseminated during their health talk. There is a need for the general public to be educated to understand the significance of the vaccination in addition to risk factors, symptoms, and screening of cervical carcinoma due to low knowledge and awareness highlighted in our study. Furthermore, community-based programs and interventional strategies must be targeted at both women and men because men were an influential element in the acceptability of the HPV vaccination by women.

Some limitations need to be highlighted. First, study participation was voluntary. Hence, most of the participants may have been those who demonstrated greater interest in the subject. Secondly, the study was limited to three cities in Ghana; therefore, the entire population cannot be generalized to our findings. Thus, the participants in the study constitute a representative sample. Also, different results will be obtained from new studies targeting rural communities and different residential areas. Thirdly, our survey centered on acceptability instead of uptake of the HPV vaccine; thus, it is uncertain if the intentions of participants to be vaccinated would turn into actions. Lastly, due to different educational backgrounds, some participants may not have completely grasped the questions, contributing to possible bias.

Taking into account the findings of this survey, certain policies can be implemented. First of all, it is important to ensure that screening facilities for cervical carcinoma are accessible in all health centers. Because of low awareness, probably providing such services in all health centers will result in an effective outreach between women and healthcare providers who may visit the health center for certain health purposes. In regards to the HPV vaccines, the higher willingness among respondents to accept the vaccine was a good sign. Since reproductive age in Ghana starts very early, it is, however, essential to begin vaccinations early in adolescence. HPV vaccines can be added to the normal vaccination program as an effective-cost solution, or “community-based vaccine drives” can be launched via the Ministry of Health. In principle, advancement in acceptance of vaccines, along with changes in behavior, would have a huge influence on Cervical Cancer Prevention in Ghana.

## Data Availability Statement

The raw data supporting the conclusions of this article will be made available by the authors, without undue reservation.

## Ethics Statement

The studies involving human participants were reviewed and approved by Zhengzhou University and Henan Provincial People's Hospital. The patients/participants provided their written informed consent to participate in this study.

## Author Contributions

ED wrote and presented the original draft. LZ and QH were involved in data curation and visualization. CE, CA, GA, ES, and FF were involved in methodology, software, analysis, review and editing. KS was involved in supervision.

## Conflict of Interest

The authors declare that the research was conducted in the absence of any commercial or financial relationships that could be construed as a potential conflict of interest.
